# Tensor decomposition for infectious disease incidence data

**DOI:** 10.1111/2041-210X.13480

**Published:** 2020-09-22

**Authors:** Hannah Korevaar, C. Jessica Metcalf, Bryan T. Grenfell

**Affiliations:** ^1^ Office of Population Research Princeton University Princeton NY USA; ^2^ Ecology and Evolutionary Biology Princeton University Princeton NY USA; ^3^ Fogarty International Center National Institutes of Health Bethesda MD USA

**Keywords:** demography, disease dynamics, measles, signal processing, tensor, wavelet

## Abstract

1. Many demographic and ecological processes generate seasonal and other periodicities. Seasonality in infectious disease transmission can result from climatic forces such as temperature and humidity; variation in contact rates as a result of migration or school calendar; or temporary surges in birth rates. Seasonal drivers of acute immunizing infections can also drive longer‐term fluctuations.
2. Tensor decomposition has been used in many disciplines to uncover dominant trends in multi‐dimensional data. We introduce tensors as a novel method for decomposing oscillatory infectious disease time series.
3. We illustrate the reliability of the method by applying it to simulated data. We then present decompositions of measles data from England and Wales. This paper leverages simulations as well as much‐studied data to illustrate the power of tensor decomposition to uncover dominant epidemic signals as well as variation in space and time. We then use tensor decomposition to uncover new findings and demonstrate the potential power of the method for disease incidence data. In particular, we are able to distinguish between annual and biennial signals across locations and shifts in these signals over time.
4. Tensor decomposition is able to isolate variation in disease seasonality as a result of variation in demographic rates. The method allows us to discern variation in the strength of such signals by space and population size. Tensors provide an opportunity for a concise approach to uncovering heterogeneity in disease transmission across space and time in large datasets.

## 1 INTRODUCTION

Seasonal and other oscillatory periodicities are widespread and important phenomena in ecosystem dynamics, climate science, health data, economic trends and many other important contexts. Seasonality is particularly critical in the study of infectious disease. Acute immunizing infections can manifest strong seasonal incidence as a result of climatic variation or periodic changes in crowding (e.g. school calendars; Dorelien, Ballesteros, & Grenfell, 2013; Ferrari et al., 2008, 2010; Grenfell, Bjørnstad, & Finkenstadt, 2002). The dynamic balance between susceptible recruitment (typically births) and herd immunity can amplify these fluctuations; often leading to long‐term fluctuations such as biennial or more exotic(multi‐annual) epidemics (Dalziel et al., 2016; Grenfell et al., 2002). Though births often demonstrate strong seasonal cycles, fluctuations in births only impact seasonality of acute immunizing infections in the absence of other forcing mechanisms (Dorelien et al., 2013). Where contact rates vary seasonally (e.g. as a result of school terms), these seasonal forces thus dominate any seasonality in births (Dorelien et al., 2013).

In the case of measles in England and Wales (E&W), and many other contexts, the predominant transmission forcing is via contact in schools (Dorelien et al., 2013; Finkenstadt & Grenfell, 2000; Grenfell et al., 2002). Effectively deterministic measles dynamics occur in large populations, where susceptible replenishment is substantial enough that infections will wane but never disappear altogether; the threshold for this epidemic equilibrium is approximately 300,000 in Europe and North America (Bartlett, 1957; Dorelien et al., 2013; Grenfell et al., 2002). In small populations, the pathogen will go extinct until it is reintroduced via imports; imported infections can trigger a large epidemic when susceptible numbers have built up sufficiently. While large locations have outbreaks at the same time every year or every other year, small towns experience more stochastic, violent outbreaks (Bartlett, 1957; Bharti, Xia, Bjornstad, & Grenfell, 2008; Bolker & Grenfell, 1995; Grenfell, Bjørnstad, & Kappey, 2001).

Wavelet analysis is used frequently to explore nonstationary cyclicality and heterogeneity in ecological time series (Bjornstad, Peltonen, Liebhold, & Baltensweiler, 2002; Cazelles et al., 2008; Grenfell et al., 2001; Grinsted, Moore, & Jevrejeva, 2004). For measles in particular, wavelets have been used to determine seasonal and longer period outbreaks as well as spatial variation in the lag between epidemics (Grenfell et al., 2001). Local dynamics of measles transmission in E&W are generally composed of seasonal (driven by the school term) and long‐term cycles usually annual or biennial driven by susceptible replenishment. The strength of these longer‐term cycles can vary between places and across time.

Wavelets provide very detailed data on seasonal signals of individual localities; in this paper, we present tensor decomposition as a method for characterizing the wavelet spectra of many places at once. Tensor decomposition is a multi‐dimensional generalization of matrix decomposition methods such as principal components analysis (PCA) or singular value decomposition (SVD). Analogous to these matrix methods, tensor decomposition reduces the dimensionality of the data by providing lower‐dimensional components which describe much of the variance in the data (Cichocki et al., 2015; Kolda & Bader, 2009; Rabanser, Shchur, & Günnemann, 2017; Sidiropoulos et al., 2017).

Tensor decomposition has been used successfully in a number of fields to uncover trends in large, multi‐dimensional datasets. The method has been used in neuroscience (Cong et al., 2012, 2015; Lee, Kim, Cichocki, & Choi, 2007; Vanderperren et al., 2013), text analysis (Acar, Camtepe, Krishnamoorthy, & Yener, 2005; Ifada, 2014; Zheng, Ding, Lin, & Chen, 2016) and photogrammetry (Guo, Huang, Zhang, & Zhang, 2013). In neuroscience, tensors have been a useful method for feature extraction and pattern detection in electroencephalography (EEG) signals. Tensor decomposition has successfully isolated task‐related brain activity from the mixture of unrelated brain activity, interference and noise (Cong et al., 2012, 2015; Lee et al., 2007; Vanderperren et al., 2013). The method can also extract differences in EEG signals among individual subjects, experimental conditions, or tasks (Cong et al., 2012; Vanderperren et al., 2013). These studies use data in some variation of a channel–time–subject format; in other words, data which can be represented coherently across three dimensions. In general, these dimensions are comprised of a subject such as an individual person or place or task, and two dimensions which represent categories (such as channels or frequencies or subject areas) and time. A data structure amenable to tensor decomposition is a one in which one dimension represents a discrete unit of observation (a trial, a person, a subject), and the other two can be easily interpreted as vectors (such as changes in power overtime across channels or frequencies). Though tensor decomposition for data structures beyond three dimensions is certainly possible, we focus on applications to three‐dimensional data both for its relative ease of understanding as well as its relevance to our project. Fields such as neurology and computer science have utilized tensors in processing signal data in a myriad of settings meanwhile applications to ecological data lag behind.

We validate the value of the method for disease incidence data by decomposing simulated epidemic data; we simulate epidemics under three different birth regimes and demonstrate the method's ability to identify these differences. We then apply the method to the well‐studied E&W measles data to uncover previously undocumented variation in measles seasonality.

## 2 MATERIALS AND METHODS

### 2.1 Simulations

We simulate epidemics using a discrete‐time stochastic susceptible‐infected‐recovered (SIR) model (Becker & Grenfell, 2017; Bharti et al., 2008; Bjørnstad & Grenfell, 2008; Caudron et al., 2014; Siettos & Russo, 2013). At each time step, each individual is assumed to be either susceptible to infection, infected or recovered (dead or immune). Once an individual recovers, we assume they can never be infected again. We simplify the simulation process by assuming that any individual infected at time *t* will be recovered at time *t* + 1.

New suscpetibles are supplied by births determined by a pre‐defined annual crude birth rate (CBR) which we distribute uniformly across each time step. Imported cases are determined by drawing from a binomial distribution with a 10% probability of importation. We use a starting population of 300,000 for all simulations, and initial infected population of 10 and initial susceptible population of 1,000. The susceptible dynamics are determined by:

(1) $$S_{t}\;=\;S_{t}\;‐\;1\;‐\;I_{t}\;+\;B_{t‐1}.$$

We add births (B*_t_*_−1_) and subtract new infections (I*_t_*). The expected number of infected individuals at time *t* is defined as a function of transmission rate (*β*), local susceptible (S) and infected individuals (I) as well as imported infections (*ι*):

(2) $$\lambda_{t}\;=\;\beta *S_{t}\;‐\;1*{\left(I_{t}\;‐\;1\;+\;\iota_{t}\right)}^{\alpha }.$$

In Equation 2, *α* is a tuning parameter, fixed to 0.97, consistent with previous analyses and simulations of measles (Becker et al., 2016; Becker & Grenfell, 2017; Grenfell et al., 2002). We give transmission a seasonal shape consistent with what has been estimated for London (Becker & Grenfell, 2017; Bjørnstad, Finkenstadt, & Grenfell, 2002; Bjørnstad & Grenfell, 2008; Grenfell et al., 2002), with an average *R*_0_ (or number of secondary infections per individual infection) set to 15 for all simulations, within the range of typical estimates for measles (Bjørnstad et al., 2002; Grenfell et al., 2002; Guerra et al., 2017). We draw the number of infections using a Poisson distribution to introduce stochasticity (Becker & Grenfell, 2017; Bjørnstad & Grenfell, 2008):

(3) $$I_{t}\;∼\;\text{Poisson}\left(\lambda_{t}\right).$$

We allow approximately 80 years for the epidemics to settle into equilibrium and evaluate the following 20 years so the scale is comparable to the 22 years of E&W data. To alter the dynamics of each epidemic, we vary the CBR used in each simulation. We use three different birth regimes: a constant CBR of 0.015, a constant CBR of 0.03, and a variable CBR which begins at 0.012 and increases to 0.036. The simulated birth rates cover the range of birth rates in the data we use; the tenth percentile of CBRs in E&W during this period is 0.012, 0.015 is approximately the median, and the max is 0.035. We collect 50 time series for each birth regime.

### 2.2 England and Wales urban districts 1944–1966

For our primary analysis, we consider measles cases in all 954 urban districts in England and Wales for the pre‐vaccination period (1944–1966) (Bharti et al., 2008; Bjørnstad et al., 2002; Caudron et al., 2014; Grenfell et al., 2001). We use annual births and population sizes to calculate the CBR for each year and evaluate the relationship between epidemic cycles and demographic conditions. In particular, we considered the CBR for locations above the critical community size for measles (Finkenstadt & Grenfell, 2000). The spatial influence of large cities on regional dynamics is substantial (Grenfell et al., 2001), and therefore we limit our analysis to larger locations as we have more confidence these locations are determining their own dynamics rather than echoing the dynamics of endemic neighbours (Bartlett, 1957; Grenfell et al., 2001). The post‐war baby boom resulted both in a surge of birth rates as well as a large range in birth rates across districts; for these reasons, we examine baby boom CBRs in particular. The following sections describe the methods (wavelet transform and tensor decomposition) we use to evaluate the relationship between epidemic seasonality and demographic conditions across all 954 locations.

### 2.3 Continuous wavelet transform

To ground our analysis in previous studies, we performed a local wavelet analysis to the log‐transformed data to assess the time‐frequency variation in the signal (Grenfell et al., 2001). Generalizing Fourier analysis, wavelets allow insight into a potentially non‐stationary epidemic signal by decomposing it into multiple frequencies over time (Gouhier, Grinsted, & Simko, 2019; Torrence & Compo, 1998). Like a Fourier Transform, wavelets decompose a complex signal into its component frequencies. However, in addition to learning which frequencies dominate the signal, wavelets allow us to determine whether and how those dominant frequencies change over time. Rather than using a sinusoid as in Fourier analysis, wavelet transformation uses wavelet basis functions which can explore local (in time) variations in frequency (Grenfell et al., 2001; Torrence & Compo, 1998). In this analysis, we used the Morlet wavelet function, essentially a damped complex exponential:

(4) $$\Psi_{0}\left(\eta \right)\;=\;\pi^{‐1/4}e^{\left(‐i\omega_{0}\eta \right)}e^{\left(‐\eta 2/2\right)}.$$

In Equation 4, *ω*_0_ is the nondimensional frequency. For a discrete sequence *x_n_*, the continuous wavelet transform (CWT) is defined as the convolution of *x_n_* with a scaled and translated version of Ψ_0_(*η*):

(5) $$W_{n}\left(s\right)\;=\;\sum_{n=0}^{N‐1}x_{n},\;\Psi *\left[\frac{\left(n^{\prime }\;‐\;n\right)\delta_{t}}{s}\right].$$

In Equation (5), (6), (*) indicates the complex conjugate. By varying the wavelet scales and translating along the localized time index *n*, one can show both the amplitude of any features versus the scale and how this amplitude changes over time. To approximate the CWT the convolution should be done *N* times for each scale, where *N* is the number of points in the time series (Gouhier et al., 2019).

**Figure 1:**
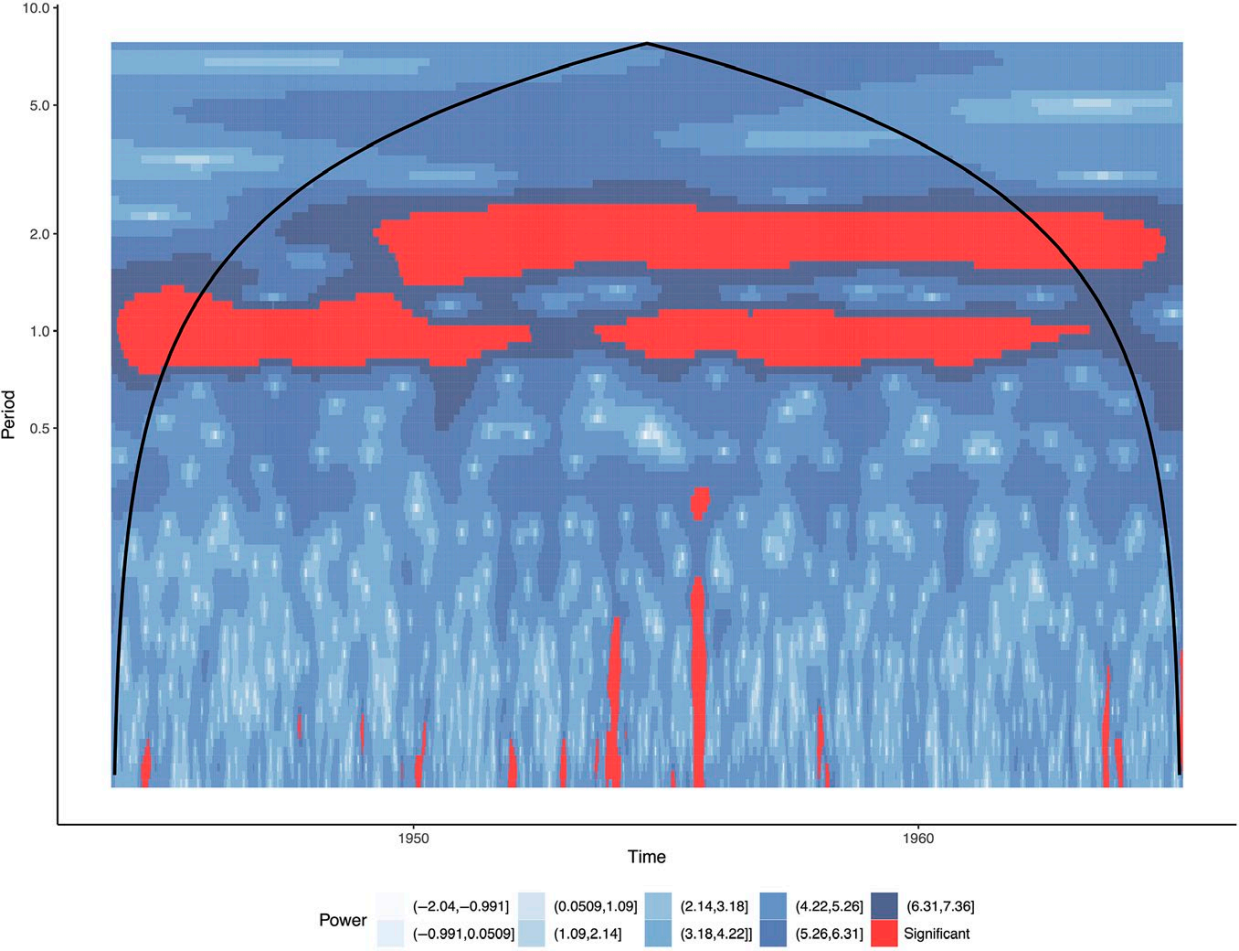
Wavelet power spectra for London (1944–1994). Darker blues indicate more power, red areas indicate time/frequencies that were determined to be statistically significant by Monte Carlo methods. The parabolic line indicates the cone of influence, the boundary of points that may be affected by edge effect artifacts. Similar to spectral analysis, errors will occur at the beginning or the end of time series. Padding the data with zeros introduces discontinuities into the data, as we increase in scale, the amplitude is decreased as more zeros enter the analysis. For the regions outside the cone of influence, it is not clear if decreases in variance are due to the additional zeros

**Figure 2:**
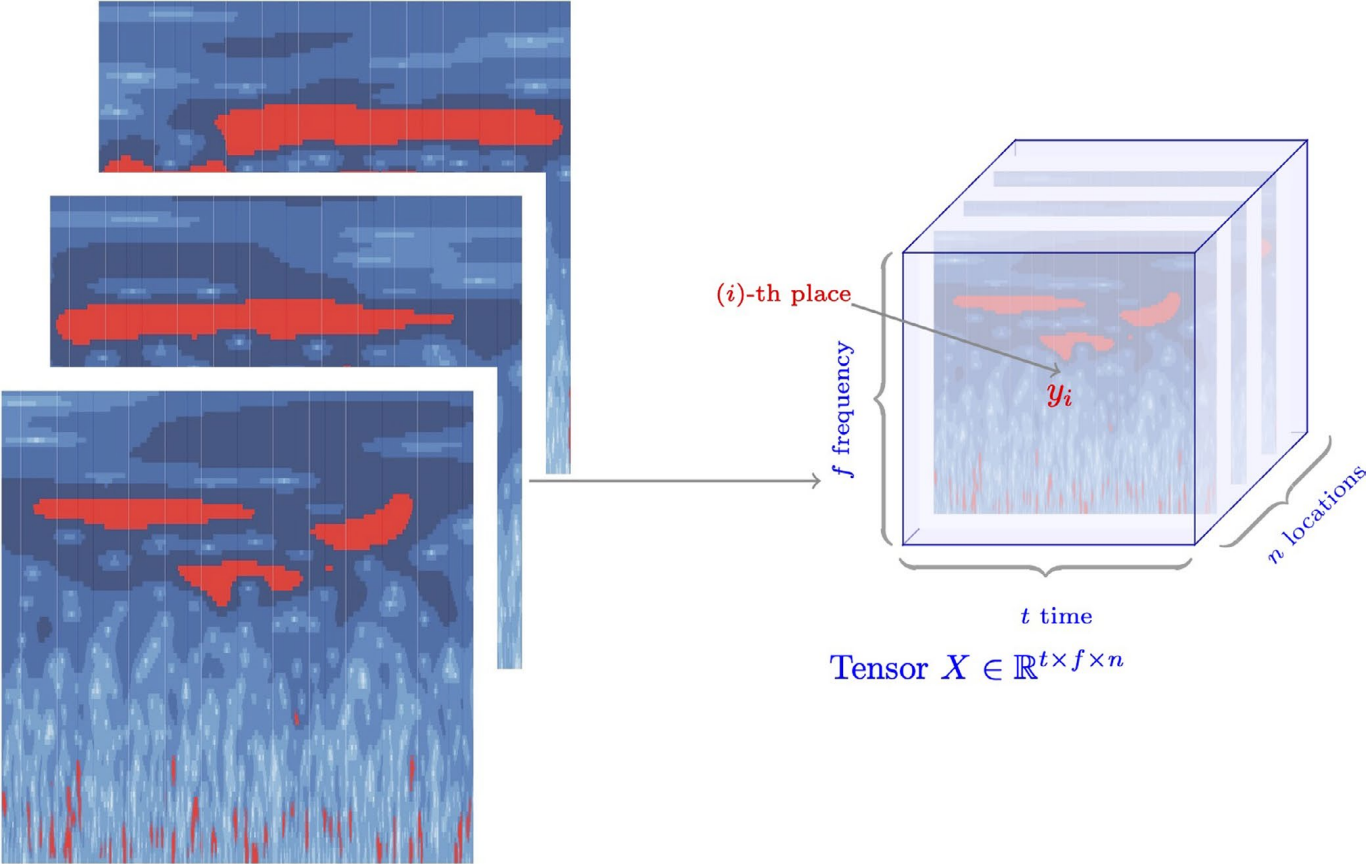
Tensor: array of wavelet spectra across locations. After calculating the wavelet power spectra, we store each of the time × frequency matrices in a three‐dimensional tensor with one matrix for each location. This creates a cube of dimensions *n* × *f* × *t* for *n* places, *t* time steps and *f* frequencies

Figure 1 provides an illustration of the wavelet power spectra for London (1944–1994). For each location (and simulation), we produce a matrix where the column indices represent time steps, and the row indices represent frequencies. The (*i, j*)‐th element in the matrix represents the power of the *i*th frequency at the *j*th time step. We then assemble these matrices into a cube by stacking them as in Figure 2.

### 2.4 Tensor decomposition

Tensor Decomposition. Once we have compiled our three‐dimensional data, we can decompose it into vector components. Tensor decomposition can be understood as a multi‐dimensional generalization of PCA (Cichocki et al., 2015; Fanaee‐T & Gama, 2016; Kolda & Bader, 2009). As with PCA, we seek to reduce the dimensionality of the data by expressing it in terms of components which capture the most variance in the data. In the CWT case, each component consists of a location vector, a frequency vector and a time vector. The outer product of the frequency and time vector produce a general wavelet power spectrum as in Figure 1. For the *i*th location, the *i*th scalar in the location vector describes the amount the power spectrum contributes to that location's original signal. In other words, each component describes a particular frequency and its power as a function of time. The location‐specific scalars represent how much that signal is magnified or dampened within that location's data.

**Figure 3:**
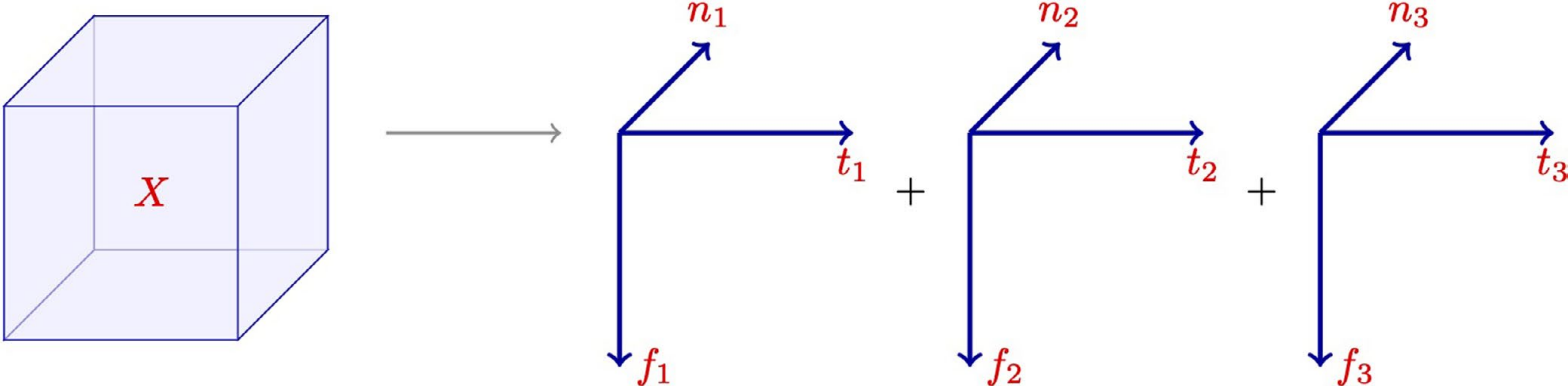
Theoretical canonical polyadic decomposition for tensor *X* from Figure 2. The outer product of each of the *f_i_* × *t_i_* vectors produces a wavelet power spectra and each of the *n* places has a score in the *n_j_* vector specific to that wavelet power spectra. In this way we can use the three rank one tensors to approximate the original power spectra for each of the *n* original places. We can think of the matrix formed by the outer product of *t_i_* × *f_i_* as a component in the principal component analysis sense. Where each entry in the matrix determines the loading of the that time, frequency value. Each entry in the *n_j_* vector represents a place‐specific score which determines the contribution of that matrix in the final wavelet power spectrum

To reconstruct the original data for a specific location, we compute and add such a matrix for each component (Cichocki et al., 2015; Guo et al., 2013; Kolda & Bader, 2009). If our tensor decomposition had three components and we wanted to reconstruct an estimate of our original data, we would calculate and sum three wavelet power spectra using the three time and frequency vectors along with the location‐specific score (Figure 3). To calculate the tensor decomposition, we use the canonical polyadic decomposition (CPD). We can formalize CPD for a three‐way tensor as follows:

(6) $$\min_{\hat{X}}||X\;‐\;\hat{X}\left|\right|\;\text{where}\;\hat{X}\;=\;\sum_{r}^{R}a_{r}\;⊗\;b_{r}\;⊗\;c_{r}\;=\;\left[A,\;B,\;C\right].$$

In Equation 6, *R* denotes the rank of the tensor (Kolda & Bader, 2009). This definition is illustrated in Figure 3 for a rank‐three tensor. We use an alternating least squares algorithm to calculate $\hat{X}$. In the case, when $\min_{\hat{X}}||X\;‐\;\hat{X}\left|\right|\;=\;0$, $\hat{X}$ is referred to as an exact low‐rank approximation of *X*. In that case, we can write out the matrix form of $\hat{X}$ as:

(7) $$\begin{array}{c} {\hat{X}}_{\left(1\right)}\;=\;\left(C\;⊗\;B\right)\;A^{T}\\ {\hat{X}}_{\left(2\right)}\;=\;\left(C\;⊗\;A\right)\;B^{T}\\ {\hat{X}}_{\left(3\right)}\;=\;\left(B\;⊗\;A\right)\;C^{T}. \end{array}$$

Each ${\hat{X}}_{\left(i\right)}$ is a component, and each *a_r_*, *b_r_*, *c_r_* are factor vectors, and *A*, *B*, *C* are factor matrices. These are analogous to components and loadings in PCA (Cichocki et al., 2015; Fanaee‐T & Gama, 2016; Kolda & Bader, 2009). To estimate these components, we fix all except one of the factor matrices and optimize the remaining matrix. For example, we may fix matrices *B* and *C* and optimize *A* given these matrices. We repeat this for each matrix until we reach our stopping criteria (Kolda & Bader, 2009; Li, Bien, & Wells, 2018). In our case, we optimize until the Frobenius norm of the error matrix is below 0.0001 (Li et al., 2018). For the three‐way tensor instance, this can be formalized as follows:

(8) $$\begin{array}{c} A\;\leftarrow \;{\text{argmin}}_{A}∥{\hat{X}}_{\left(1\right)}\;‐\;\left(C\;⊗\;B\right)\;A^{T}∥\\ B\;\leftarrow \;{\text{argmin}}_{B}∥{\hat{X}}_{\left(2\right)}\;‐\;\left(C\;⊗\;A\right)\;B^{T}∥\\ C\;\leftarrow \;{\text{argmin}}_{C}∥{\hat{X}}_{\left(3\right)}\;‐\;\left(B\;⊗\;A\right)\;C^{T}∥. \end{array}$$

Characterizing the rank of a tensor is a complex mathematical problem without a simple solution (Alexeev, Forbes, & Tsimerman, 2011; Ballico, Bernardi, Chiantini, & Guardo, 2018; Kolda & Bader, 2009; Stegeman & Friedland, 2017). Therefore, we attempt tensor decomposition beginning by selecting a single component, and increasing the number of components until the algorithm consistently converges.

## 3 RESULTS

### 3.1 Simulations

**Figure 4:**
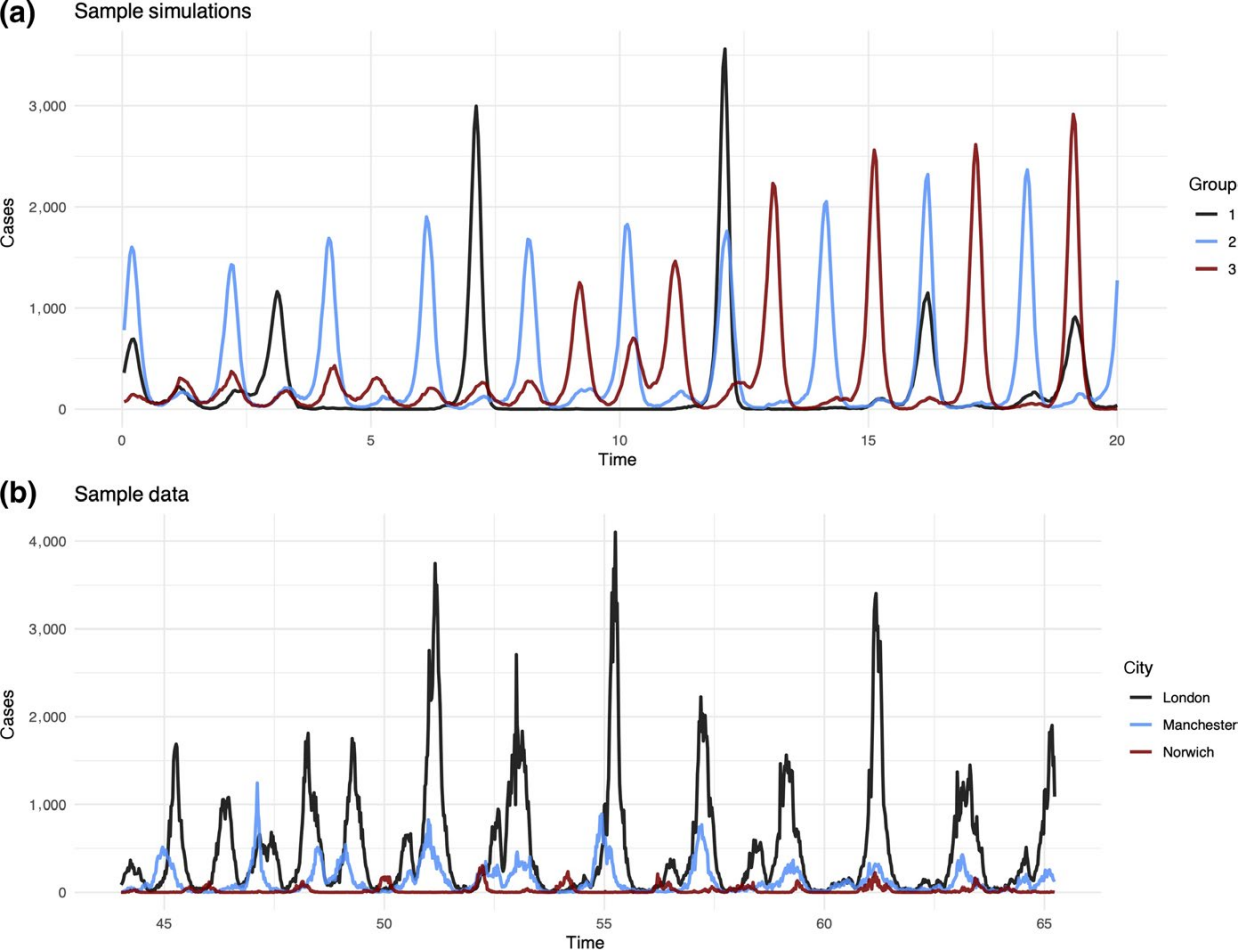
Panel (a) shows a sample of simulations generated under different birth regimes. Group one is constant CBR of 0.015, group two is constant CBR of 0.03 and group three is CBR beginning at 0.012 and growing to 0.36. We see that group three begins with dynamics similar to those of group one; by the end of the 20‐year period, group three more closely resembles group two. Panel (b) shows case data from three cities of different cities and periodicities from the E&W data

**Figure 5:**
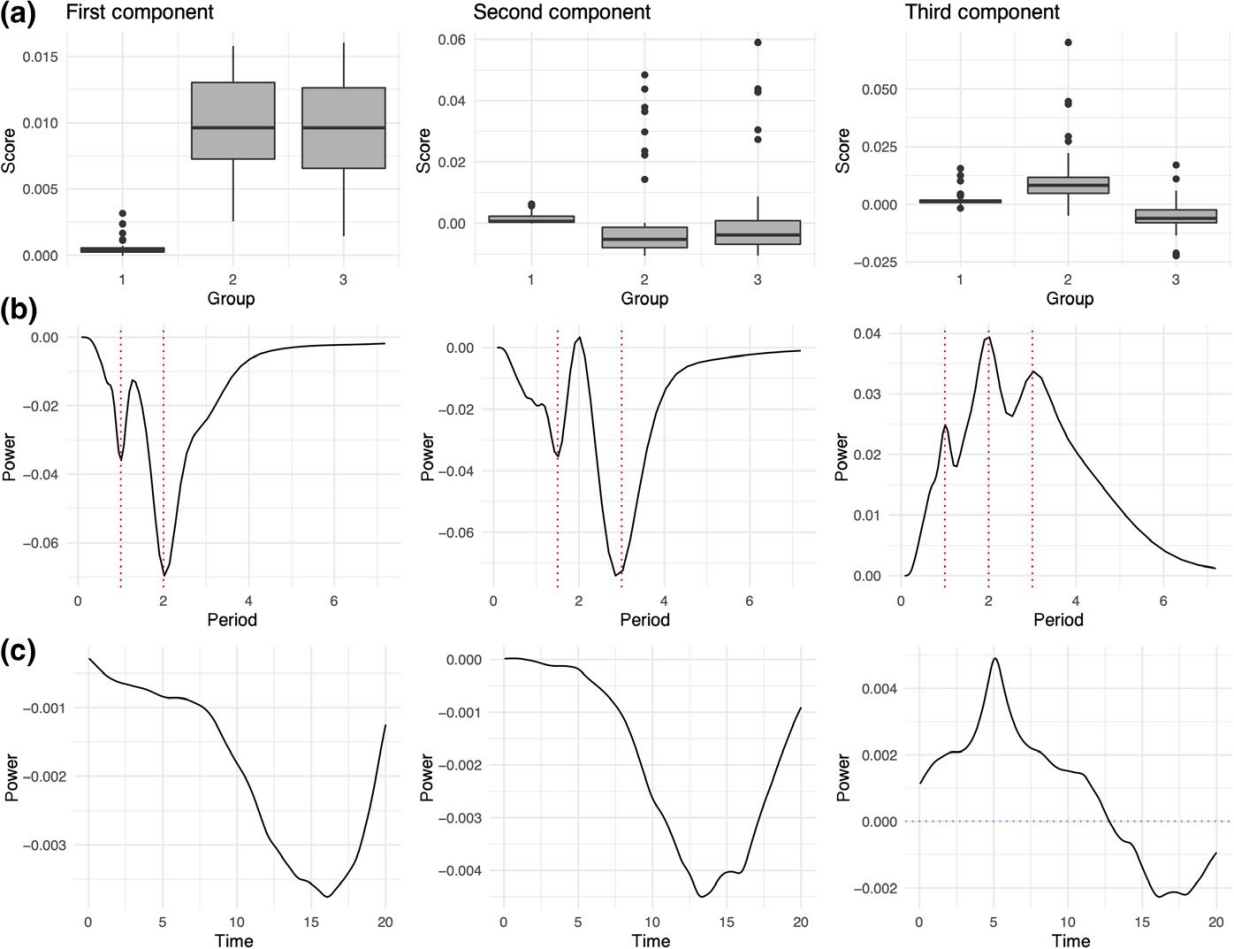
Three‐rank tensor decomposition of the 150 simulated places. Panel (a) shows the distribution of scores on each of the three components for each of the three groups. Panel (b) shows the dominant periods explained by each component. Panel (c) shows the temporal trends for the periods depicted across panel (b). Each column gives a full description of each of the three components

Our simulations demonstrate comparable epidemic behaviour to that of E&W (Figure 4). The CPD algorithm consistently converged at three components for the simulated data. In Figure 5, each column corresponds to a component of the decomposition, each row represents the dimensions of the tensor: simulation group (A), period (B) and time (C).

The first row of Figure 5 shows the variation in score on each component by group. The third component is able to distinguish group two (higher birth rate) from group three (increasing birth rate). This component identifies a temporal shift where 1‐, 2‐ and 3‐year periodicities shift in strength from the beginning to end of the time series (Figure 5, panel c). The power of the signal peaks in the first quarter, changes sign at halfway, and reaches a nadir in the final quarter. A positive score on the third component indicates stronger signals in the first half of the time series and a reduction in the second half. A negative score on this component would indicate the opposite—weaker signals in the first half of the time series and increasing 1‐, 2‐ and 3‐year periodicities in the second half.

Note that the period and time vectors for both the first and second component are negative, which means their product is positive. The first component therefore demonstrates an increase in biennial and annual signal over time, the second component describes an increase in 1.5‐ and 3‐year signals over time.

Adding components with group specific scores results in a consistent annual and triennial periodicity for the first group (low birth rate); consistent annual and biennial cycles in the second (high birth rate); and a transition from sporadic epidemics to annual and biennial signals in the third group (increasing birth rate). These results concur with samples of the time series (Figure 4) as well as reconstructions of the CWT for each group (Figure S1).

### 3.2 England and Wales urban districts 1944–1966

**Figure 6:**
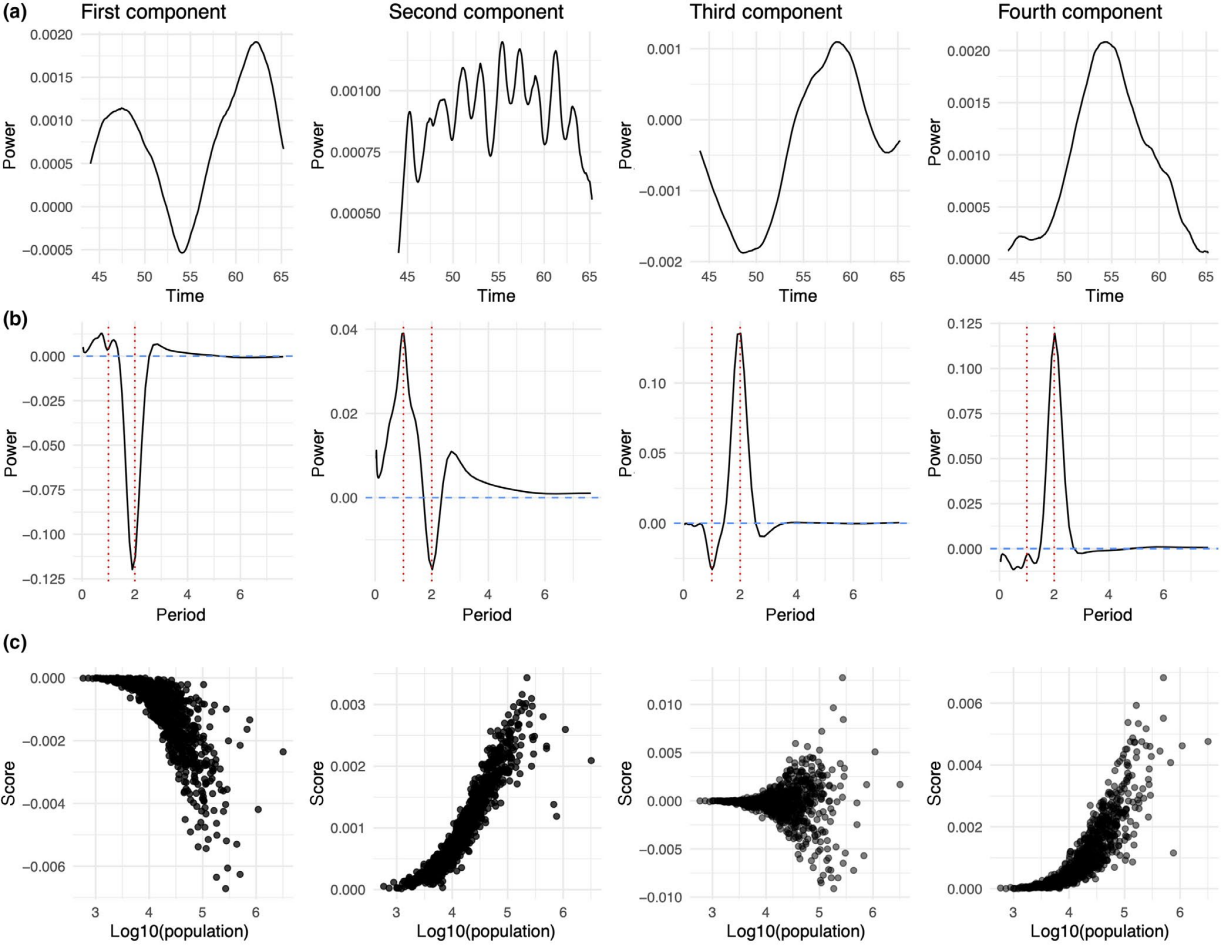
The four components of the tensor decomposition for E&W urban districts. Panel (a) shows each of the time components, panel (b) shows each of the period components and panel (c) shows the distribution of scores on each component by population size. Each column corresponds to a single component (e.g. the first column represents the time, period and district‐specific scores for the first component of the decomposition). We see that small districts consistently score near zero on each component. This is consistent with previous work which suggests dynamics in small districts are highly irregular and lack the seasonal signature we see in large districts

We found four components provided the most consistent convergence with the CPD algorithm for the E&W tensor (Figure 6). The most succinct way of classifying variation in measles dynamic across the country is variation in the strength of the annual component throughout the 1944–1965 time period, and whether the biennial component peaks at the beginning or the end of the era.

The dominance of the annual and, in particular, the biennial signal in these data has already been documented (Grenfell et al., 2001). If we were naive regarding the importance of these signals, we could investigate variation in biennial patterns regionally—including synchronicity and temporal lag. Bjorn tad and Grenfell have investigated such patterns in this dataset (Grenfell et al., 2001).

**Figure 7:**
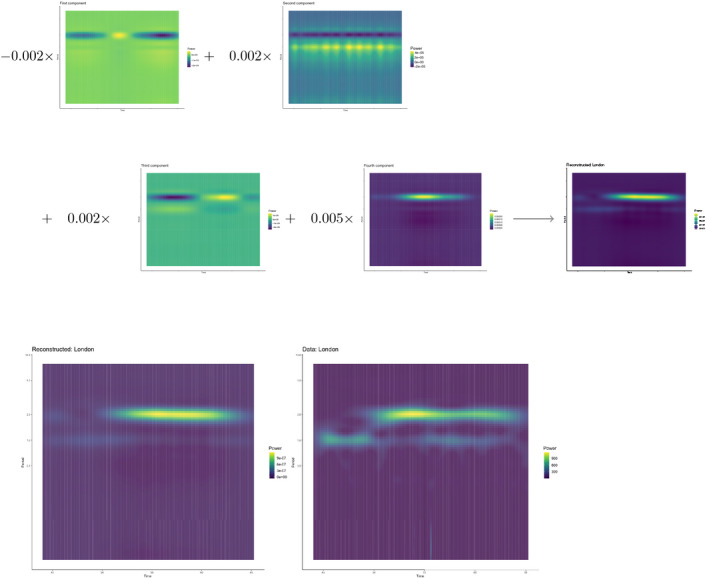
A component by component illustration of the reconstruction of the London wavelet spectra. Each time‐frequency component is multiplied by the location‐specific scalar. These scalars can amplify signals or switch their signs and determine the influence of each component on the final reconstruction. The bottom row compares the original with the reconstructed spectra

**Figure 8:**
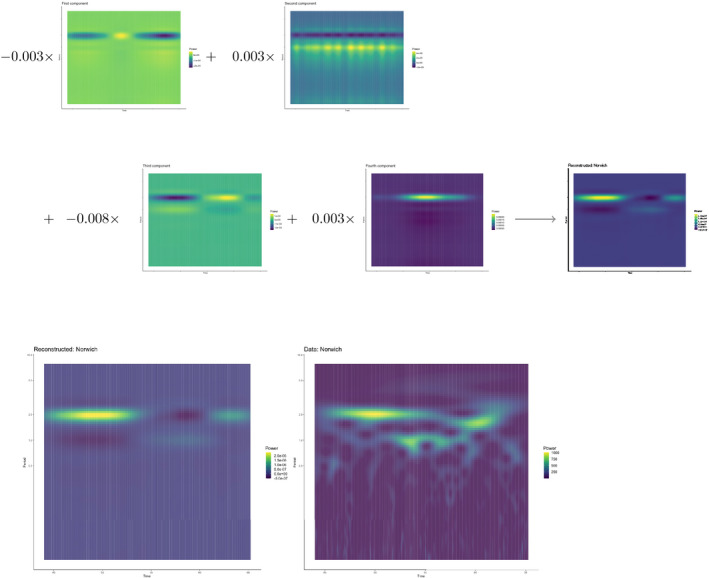
A component by component illustration of the reconstruction of the Norwich wavelet spectra. Each time‐frequency component is multiplied by the location‐specific scalar. These scalars can amplify signals or switch their signs and determine the influence of each component on the final reconstruction. If we compare this reconstruction to the London reconstruction, we can see how adjusting sign and magnitude of the scalars alters the reconstructed frequency‐time spectra. The bottom row compares the reconstructed with the original spectra

To validate the decomposition for such a large dataset, we reconstructed the time series for four districts with distinct dynamics (Figure S3). An illustration of the reconstruction for London (Figure 7) and Norwich (Figure 8) shows how each component contributes different frequency‐time power. These reconstructions demonstrated the ability of a few components to explain a large amount of time‐frequency variation.

The strength of the score on all components depends largely on population size. This is consistent with previous work which has shown that dynamics in small, isolated places tend to be erratic rather than seasonal and thus do not have significant annual or biennial signals (Bartlett, 1957; Bjørnstad & Grenfell, 2008). Though small places consistently score near zero on each component, they generally have the largest scores on the second component relative to the others. Positive, nonzero scores on this component alone is consistent with irregular epidemics. As large districts tend to have higher magnitude scores on the other components, the influence of this irregularity component is reduced for those locations.

The third component uncovers the most variability in the dynamics of large places. Here negative values are associated with early (pre‐1955) biennial signals and late (post‐1955) annual signals. Positive values predict the opposite: early annual signals and late biennial signals. While all other signals tend to increase in magnitude with population size, the third component increases both in magnitude with varying sign, indicating a substantial deviation in the signal of large places. In addition to variation in the sign of the scores on this component, the magnitude of the scores is greater by nearly a factor of 10. We investigate this component further as it indicates an important dovetailing of epidemic dynamics across locations.

We examined CBRs over the entire time period as well as CBRs in the first four years of the time series. E&W experienced a post‐war baby boom between 1944 and 1948 which peaked in 1947. We took the average of the birth rates between 1944 and 1948. We find when we average CBRs across the 20‐year time period, we see little variation across locations. Baby boom CBRs have much greater variability. We see a statistically significant difference, where higher baby boom birth rates correspond to positive scores on the third component. This indicates locations with higher birth rates at the beginning of the time series have stronger annual signals early in the time series compared with their lower birth‐rate counterparts (Figure S2). We know from mathematical models of measles transmission that higher birth rates should lead to larger annual epidemics, as a result of quicker susceptible replenishment. In concordance with these models, our decomposition shows that locations with slightly lower CBRs would begin with more biennial seasonality and locations with crude higher birth rates would begin with more annual seasonality and settle into biennial cycles later. The baby boom CBR has been identified as a crucial bifurcation point for measles cycles using simulations (Finkenstadt & Grenfell, 2000). This local dovetailing in dynamics as a result of variation in the CBR has not been previously illustrated.

## 4 DISCUSSION

In this paper, we explore, to our knowledge, the first application of tensor decomposition to disease surveillance data to confirm previous findings regarding (a) the dominance of annual and biennial signals in the time series across locations; (b) the deterioration in the signal of epidemic seasonality in small populations and (c) the importance of crude birth rate in local dynamics using all 954 urban locations. We have also shed novel light on the well‐known importance of baby boom births in local dynamics.

Tensor decomposition shows promise in its ability to distil multi‐dimensional data into lower dimensional components. Though tensors have been used in many fields of research, their applications to epidemiological data are still under‐explored. Here, we use tensor decomposition to reveal heterogeneities in time, space and frequency. Since many of the differences illustrated here are well documented in previous studies of this data, we are confident in the ability of tensor decomposition to uncover the dominant trends in the time series.

We have demonstrated the utility of tensor decomposition for infectious disease data; more broadly, this method is applicable to any spatiotemporal cyclical phenomena in ecology or population science. Though we have only touched on all its applications here, this method can (a) reconstruct original signals without additional noise; (b) concisely summarize dominant trends and heterogeneities in an otherwise unwieldy dataset; (c) identify the appropriate frequencies at which to evaluate phase differences, synchrony, and lag.

Figures 7 and 8 demonstrate the ability of tensor decomposition to capture variation across dominant signals in the data. In the case of this paper, these components focus on the shifts in annual and biennial signals. In these constructions, we see the backbone of the original signals with much of the noise removed. However, there may be cases in which this succinct representation is not desirable. For example, one may be interested in the frequencies which were dropped, such as 1.5‐year or 3‐year cycles. Tensor decomposition, like PCA, will select the most efficient representation of the data; inherent to this method is the loss of some information. However, there are solutions which can increase the granularity of the components.

As with PCA, one can opt to decompose the data into additional components. We selected four components for this paper because the CPD algorithm reliably converged at four components, and because each additional component adds substantial computation time to the decomposition algorithm. For these data, if one were interested in more detailed information, one could select a subset of the total dataset which would allow for faster computation of additional components. This subsetting could be done by location (e.g. a random sample or a sample of the largest, most dominant cities), or by frequency (e.g. selecting all data between annual and triennial frequencies).

A first pass naive tensor decomposition, such as the one presented in this paper, may guide additional decompositions. In the case of Norwich (Figure 8) that the 1.5‐year cycle in the data is represented as a 2‐year cycle in their construction. After a first pass tensor decomposition, the data could be pre‐reduced by dropping frequencies. In this case, the data for 6‐month periods and below accounts for very little variance in the entire data. Dropping these frequencies could allow the representation of additional cycles in the tensor components, as well as reducing overall computation time.

An additional extension of this analysis would be to use tensor decomposition on the phase or phase‐differenced matrices for these locations in order to concisely summarize spatiotemporal dynamics of epidemics (Grenfell et al., 2001).

## AUTHORS' CONTRIBUTIONS

H.K. conceived of the initial project design, carried out the analysis and drafted the manuscript; C.J.M. and B.T.G. provided project guidance, suggested additional project components and provided editorial feedback on the manuscript.

### PEER REVIEW

The peer review history for this article is available at https://publons.com/publon/10.1111/2041‐210X.13480.

## Supporting information

Supplementary Material

## Data Availability

The measles data used in this paper are publicly available via https://www.nature.com/articles/s41559‐020‐1186‐6 (Lau et al., 2020) (see references). The code used to simulate disease data, calculate wavelet transform and decompose the tensor is available https://doi.org/10.5281/zenodo.3999553 (Korevaar, 2020).
