# Supplementary material for: Tensor decomposition for infectious disease incidence data
Source: Methods Ecol Evol. 2020 Sep 22;11(12):1690–700. doi: 10.1111/2041-210X.13480 (PMC7756762; doi:10.1111/2041-210X.13480)
Supplement: Supplementary file 1 — Supplementary Material [file MEE3-11-1690-s001.docx]

Tensor Decomposition for Infectious Disease Incidence Data: Supplementary Material

Supplement Figure 1: Averaged and reconstructed power spectra for simulated data. We see a large amount of variation as a result of stochasticity within each group. However, we do see constant annual power in each location. We see the biennial signal becomes stronger over time in the third (variable birth rate) group. Additionally we see more long-term cycles in the first (low birth rate) group. We see the reconstructed power spectra are able to pick up much of this variation


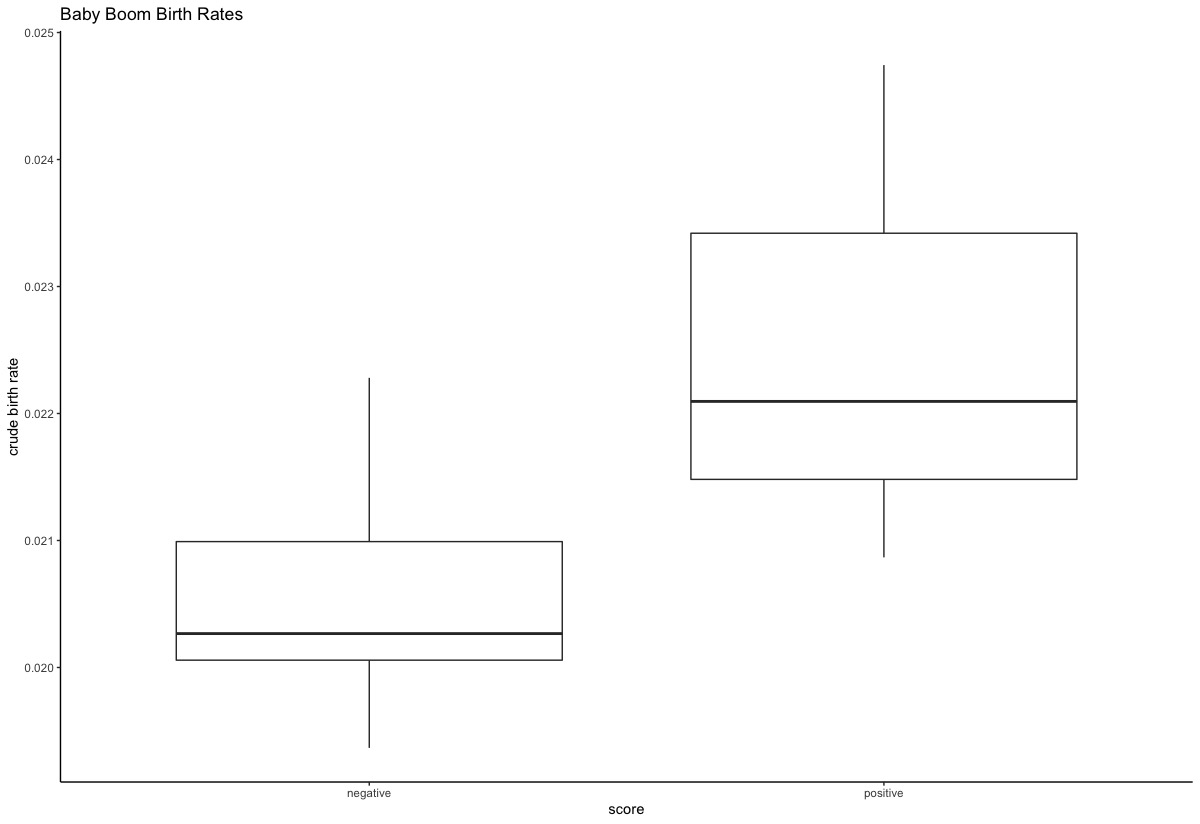


Supplement Figure 2: Crude Birth Rates for locations above the critical community size in England and Wales grouped by positive and negative scores on the third component of the tensor decomposition. We see that locations with higher crude birth rates are more likely to have a positive score on this component, indicating larger annual epidemics early in the time series.

Supplement Figure 3: Reconstructed and original power spectra for (A) London, (B) Manchester, (C) Norwich, and (D) Leeds. We can see that the four components are able to capture variation in the timing of annual and biennial periodicities. We also see that much of the noise and idiosyncrasies of individual time series is reduced. In other words, the reconstructions pull out the dominant signals and enable a comparison across these patterns
